# Assessment of soil- and water-related ecosystem services with coupling the factors of climate and land-use change (Example of the Nitra region, Slovakia)

**DOI:** 10.1007/s10653-023-01656-y

**Published:** 2023-06-21

**Authors:** Fatemeh Adelisardou, Peter Mederly, Tatian Minkina

**Affiliations:** 1 Department of Ecology and Environmental Sciences, Faculty of Natural Science and Informatics, Constantine the Philosopher University in Nitra, Nitra, Slovakia; 2 Department of Soil Science and Land Resources Estimation, Southern Federal University, Rostov-On-Don, Russia

**Keywords:** Ecosystem services, Climate change, Land use change, Hotspot, Coldspot, InVEST model

## Abstract

Climate and land use change can profoundly impact the provision of ecosystem services (ES) over time, particularly in the landscape of open fields along with growing urbanization and rising demand for space, food and energy. Policymakers are keen on knowing the combined effects of climate and land use change on ESs as a critical issue in human well-being. However, deep knowledge of how to identify these relationships is still lacking. This research aims to undertake a comprehensive assessment of soil- and water-related ES, and improvement in understanding how they are affected by climate and land use change. We applied the Integrated Valuation of Ecosystem Services and Trade-offs model for four ES (soil retention, nutrient delivery ratio, carbon storage, and water yield) for the years 2000 and 2018 in the Nitra region, Slovakia. We investigated the spatial and temporal changes in ES provision and determined the hotspots and coldspots of multiple ES. We found that soil retention, water yield, and carbon storage display a rising trend while the nutrient delivery ratio showed a decreasing trend over the past 18 years. Although all the mentioned services mainly attributed to land use change, the relative contribution of climate change was not deniable. Forests in the north and east and distributed urbanization and agriculture are the hotspots and coldspots for all ESs, respectively. Our results, in terms of determining the relative importance of land use and climate change and identifying the sensitive areas of ES provision, provide a scientific basis for ecosystem conservation and management priority setting at the local and regional levels.

## Introduction

Growing urbanization at an exponential rate, increasing demand and change in human activities have accelerated the change in world landscape patterns (Chen et al., 2020). Ecosystem degradation, which in turn threatens human well-being (Lyu et al., 2018) and sustainable structure and function on different scales are the most significant results of this trend (Lyu et al., 2018; Zope et al., 2016). The mentioned trend is strongly connected to the environmental paradox of correlation between increasing human well-being and ecological degradation. Ecosystem balance and a good state of health is essential for resilience in environmental stresses(Ma et al., 2022a, b) maintaining human survival (Chen et al., 2023; Qiao & Huang, 2022) and well-being(Bao et al., 2022; Zou et al., 2022).

Ecosystem health is broadly defined as the state or condition of an ecosystem in which its dynamics are expressed within a normal range of activity based on its level of ecological development (Andel and Aronson, 2006). This has led to increased research into the study of ecosystem services (ES) and their related fields of ecosystem health and environmental sustainability (Qiu et al., 2022; Ward et al., 2018). ES encompass the multitude of benefits that humans receive from ecosystems, which are further divided into four categories: support, regulation, provision, and culture (Costanza et al., 2017). Results from the Millennium Ecosystem Assessment demonstrate that at least 15 (ES), such as water purification and erosion regulation, have experienced a decrease in their provision capacity. This trend could potentially worsen in the coming years (Kubiszewski et al. 2017). Considering the multiple trade-offs associated with the provision of services, the flux of ESs bundles can be effectively managed (Howe et al., 2014). For instance, crop production causes tradeoffs with other ES, such as water quality(Qiu et al., 2022) or carbon storage (Adelisardou et al., 2021). Regular monitoring of ES is essential to gain a better systematic understanding of their status, distribution, and changes over space and time, particularly in the Anthropocene(J.-M. Gao et al., 2017a, 2017b; Hekmatfard et al., 2013). ES are affected through multiple drivers including climate change (Cornell et al., 2019; Leal Filho et al., 2021), resource degradation (Weerahewa et al., 2023), population growth (Reed et al., 2015) and land use change (Lang & Song, 2019). The improper use of resources and lack of consideration of sustainability in the management policy could lead to the depletion of essential services for future generations (Lang & Song, 2019).

Studies have identified climate change and land use modifications as the key forces altering the equilibrium, unpredictability, and dynamism of ES over time(Ma et al., 2022a, b; Oh & Yoon, 2022). The quantity and quality of ES provision over time are being threatened by climate and land use change as a coupled driver(Li et al., 2021). Specifically, a strong correlation has been identified between the distribution of particular land cover and climatic factors. Based on Song et al., 2018 approximately 60% of land use change is associated with human activities and the rest is related to the other driving forces such as climate change. The structure, function, and services of ecosystems are greatly altered by climate change due to its influence on biochemical and hydrological processes (Weiskopf et al., 2020). For instance, water scarcity during drought periods can alter crop production, soil quality and water treatment cost through increased evaporation. It also impacts the social-ecological systems, individual species and their interaction with other organisms in their habitats (Postigo, 2021). Land use change as the next significant driver has a great influence on the structure, function of ecosystems and their capacity to provide ES (). A decreasing trend in the supply capacity of nutrient flow and carbon balance in urban areas, which is attributable to land use change, is evidenced(Cornell et al., 2019).

Investigation of the responses of ES to climate and land use change has enabled the formation of a more comprehensive understanding of the implications of these changes for policy-making and conservation strategies. With such knowledge, decision-making, and policies regarding the sustainable management of ecosystems and societal relations can be better evaluated and informed. Several studies have been conducted on this topic, each emphasizing different factors. Some have examined the effect of land use changes on ES independently (Adelisardou et al., 2021; Gao et al., 2017a, b; Nijhum et al., 2021; Wang et al., 2017). Others have focused on the impacts of climate change (Manes et al., 2022; Saeed et al., 2022). The influence of urbanization on ES has also been a subject of study (Luiza Petroni et al., 2022), as have the unique challenges and conditions presented by coastal ecoregions (Lheureux et al., 2023), arid islands (Li et al., 2021), and river systems (Zhang et al., 2022a, b). Each of these investigations contributes to a broader understanding of how different factors shape the responses of ES to change, however, quantitative studies on the effects of their combination on ESs are still insufficient.

This study bridges existing knowledge gaps by assessing the spatiotemporal responses of soil- and water-related ES in Nitra, Slovakia to climate and land use change over the period 2000–2018. We employ a novel coupled modeling-mapping approach to accurately assess the connections between ES and drivers of change, and the findings of this study are of international validity. The research delivers appropriate indicators to aid the identification and implementation of proper practices to manage ES budgets at local and regional levels. By providing a more detailed understanding of the interactions between climate change, land use, and urbanization, our results have general relevance, adding to international knowledge and capabilities for adaptive responses to future changes in a wide range of locations.

We studied four services provided by the Nitra region's landscape system: soil retention, nitrogen export, carbon storage, and water yield, over time. Agricultural activity has caused widespread, negative effects, and its impact on the quality of these services has had major implications for the health of neighboring ecosystems. We utilized the InVEST software tools to model ES by analyzing historical trends from the past to the present. Our study quantified the impacts of climate and land use change on ES provision and addressed the following questions: (1) how did the four mentioned ES change in Nitra region, Slovakia over the 18 years, and (2) what was the relative importance of climate and land use change in determining shifts in the mentioned ESs?

## Materials and methods

### Study area

**Fig. 1:**
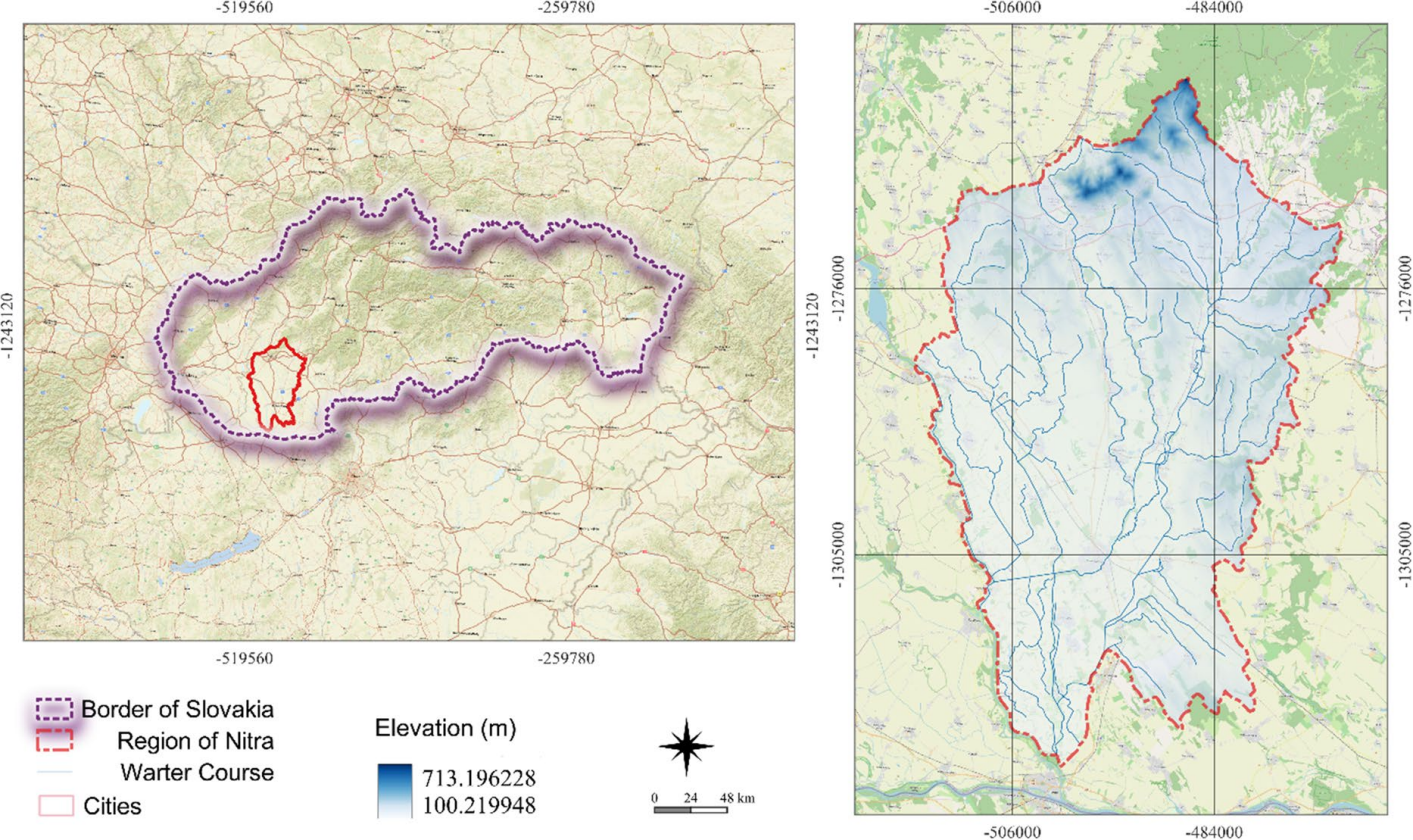
The geographical location of Nitra region, Slovakia

The study area of the Nitra region, located in the southwest of Slovakia and part of the Nitra NUTS3 region, encompasses six districts. The study area covers 4.2% of Slovakia's total area and consists of 146 municipalities with a population of 298,500 (5.5% of the country's total population), with Nitra (77,600 inhabitants) and Nové Zámky (37,300 inhabitants) being the largest cities. It is found in the catchment basins of the Nitra and Žitava Rivers and covers 2,070.6 km2 (Fig. 1). 9.6% of the area is urbanized. The remaining land is in the Podunajská nížina lowland, with 77.5% of the area used for productive agricultural land, mostly for arable crops. The northern part of the area is located within the Tríbeč mountain range, where the predominant land cover is forest, accounting for 8.8% of the area.

## Research framework of assessing the impacts of climate and land use change on selected ES

**Fig. 2:**
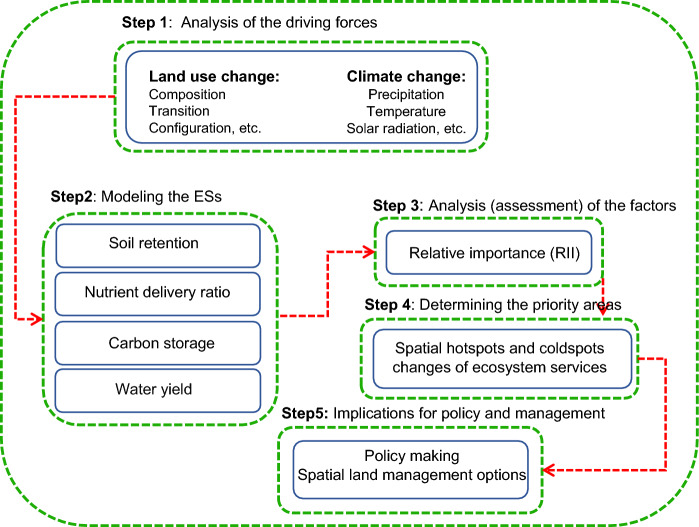
The general workflow of research procedure

Five steps were identified to explore how climate and land use changes affect ES in Nitra, Slovakia. The goal of this framework is to guide optimal land management decisions (Fig. 2). In this study, we characterized two forces of potential change (climate and land use) based on available data and literature. Moreover, we employed InVEST to model four ES for two years (2000 and 2018). In the third step, we examined how climate change and land use affected changes in the values of ESs. Subsequently, in step four, we identified areas of high and low ES values. Policy makers and planners should consider the results of this study to develop strategies that better manage environmental sustainability.

### Climate and land use data and processing

We compared 2000 and 2018 Corine Land Cover layers (CLC) to identify changes in seven primary land cover categories—developed areas, arable land, orchards and vineyards, woodlands, grasslands, forests and water bodies. We used the Semi-Automatic Classification Plugin (SCP) Postprocessing Tool (QGIS 3.26.3) to compare the land use maps and quantify land use changes over the 2000–2018 period. As climate data, for 2000 we used precipitation and temperature maps from the Climate Atlas of Slovakia (https://climate-adapt.eea.europa.eu), representing the long-term average values of these parameters. For 2018, we changed these values based on determined trend-lines resulting from time series of precipitation and temperature data for the 1990–2020 period. The data sources are summarized in Table 1.

**Table 1:** Data requirement for the InVEST models

Data	Type	Data source	Note	Related model
Digital Elevation Model	Raster	https://www.eea.europa.eu/data-and-maps/data/copernicus-land-monitoring-service-eu-dem	EU-DEM; resolution 30 m × 30 m	NDR, SDR
Annual average precipitation	Raster	Climate Atlas of Slovakia: https://www.shmu.sk/en/?page=2169	Resolution 30 m × 30 m	WY, NDR, SDR
Reference evapotranspiration	Raster	Climate Atlas of Slovakia: https://www.shmu.sk/en/?page=2169	Resolution 30 m × 30 m	WY
Plant available water content	Raster	https://websoilsurvey.nrcs.usda.gov	Resolution 30 m × 30 m	WY
Land use / land cover	Raster	https://land.copernicus.eu/pan-european/corine-land-cover	LULC of year 2000 and 2018, resolution 30 m × 30 m	WY, NDR, SDR, CS
Depth to root restricting layer	Raster	https://websoilsurvey.nrcs.usda.gov	Resolution 30 m × 30 m	WY
Watersheds	Vector		A shapefile determined by DEM raster using ArcGIS tool	WY, NDR, SDR
Rainfall erosivity index	Raster	https://esdac.jrc.ec.europa.eu/content/global-rainfall-erosivity	Resolution 30 m × 30 m	SDR
Soil erodibility	Raster	https://websoilsurvey.nrcs.usda.gov	Resolution 30 m × 30 m	SDR
Biophysical tables	.CSV file	InVEST models tutorials	Including attributes of each LULC, carbon pools and etc	WY, NDR, SDR, CS

Water yield model = WY; Nutrient delivery ratio model = NDR; Sediment delivery ratio model = SDR and Carbon storage model = CS

### Modeling of ecosystem services

InVEST (Integrated Valuation of Ecosystem Services and Tradeoffs) is a suite of models used to map and value different natural services that help sustainable human well-being (Peng et al., 2020). As the Nitra region is a hotspot of agricultural activities and crop production in Slovakia, four significant soil- and water-related services including soil retention, nutrient delivery ratio, carbon storage and water yield were selected. In this regard, this study used the InVEST model (version 3.12.0) for modeling the above-mentioned services for two different years of 2000 and 2018. The modeling procedures are described in the following subsections.

### Soil retention

In the InVEST Sediment Delivery Ratio (SDR) model, the proportion of soil loss that reaches the catchment outlet is computed by first quantifying the amount of sediment generated from erosion and then calculating the ratio of the eroded sediment that is delivered to the streams. (Sharp et al., 2016). The general inputs of the SDR model are land use / land cover map for the specific time, precipitation, digital elevation model, and soil erodibility. This model also requires a biophysical table associated with the land cover classes. The expected outputs from the SDR include annual sediment loads to streams, the amount of sediment eroded in the catchment scale, and finally the amount retained by features such as topographic and vegetative cover.

### Nutrient delivery ratio

The model of Nutrient Delivery Ratio (NDR) in InVEST models the source of nutrients from watershed and the amount of nutrients transported to the streams (Sharp et al., 2016). The data needed for the purification model include land use/land cover map, precipitation, DEM, and also a biophysical table associated to the land cover map. This model describes the movement of nutrient mass using a balanced approach through space. The load of each pixel is modified based on the potential of runoff at the local scale that could be divided to subsurface and surface runoff (Sharp et al., 2016).

### Carbon storage

The carbon model of InVEST uses a simplified carbon cycle to estimate the amount of static carbon storage and dynamic sequestration for each cell in a specific region (He et al. 2016). This model considers four carbon pools, including aboveground carbon density (C_ above), belowground carbon density (C_below), soil organic carbon (C_soil), and dead organic matter (C_dead) (Tallis et al. 2013). The needed data for running the carbon storage model include land use/land cover map and the biophysical table containing columns of land use, ‘C_ above,’ ‘C_below,’ ‘C_soil,’ and ‘C_dead.’

### Water yield

The average and summary water yield could be estimated in the water yield model of InVEST(J. Gao et al., 2017a, 2017b). The mean amount of water that runs off through the landscape is defined as water yield. Considering the water balance at the sub-watershed scale is one of the principles of this model (Gao et al., 2017a, 2017b). The model necessitates the incorporation of precipitation, reference evapotranspiration, land use map, depth to root restrictive layer, a plant available water fraction layer, and biophysical table data as inputs.

### Processing of the results

#### Climate and land use changes settings

In this study, we evaluated the combined effect of land use and climate change on ES in Nitra between 2000 and 2018. We considered changes in observed climatic data and land use within the study region, allowing us to examine spatiotemporal patterns of changes in environmental conditions and ES. This approach allowed us to identify the extent to which each driver contributed to variability in ES.

### Relative importance analysis

We used the Relative Importance Index (RII) proposed by Su et al. (2022) to analyze the effects of climate and land use change on ES in the Nitra region. The RII was calculated for each ES over the entirety of the study area and portrays the relative influence of each driver on the respective ES for each pixel. RII values higher than 0 indicate that land use change has greater impacts than climate change on the ES, while values less than 0 signify the importance of climate change. A value of 0 indicates equal influences of both drivers on the ecosystem services.

### Hotspots and coldspots analysis

We attempted to identify the hotspots and coldspots in the provision of bundles by examining the values of all four ES. ArcGIS's Getis-ord Gi* statistics tool was used to detect statistically significant hotspots. This tool accurately identifies regions of high (hotspots) and low (coldspots) values within a grid, providing valuable information that can be used to prioritize conservation efforts (Li et al., 2017a, 2017b). Areas with higher z-scores and lower p-values show statistically significant hotspots, while those with lower negative z-scores and smaller p-values reveal statistically significant coldspots. This methodology can accurately identify areas of both high and low significance, thus enabling stakeholders to prioritize their management tasks according to the actual demands.

### Used data overview

Table 1 summarizes the data used for running the ES models, including their source, type, resolution, and purpose.

## Results

### Land use and climate changes

#### Land use

**Fig. 3:**
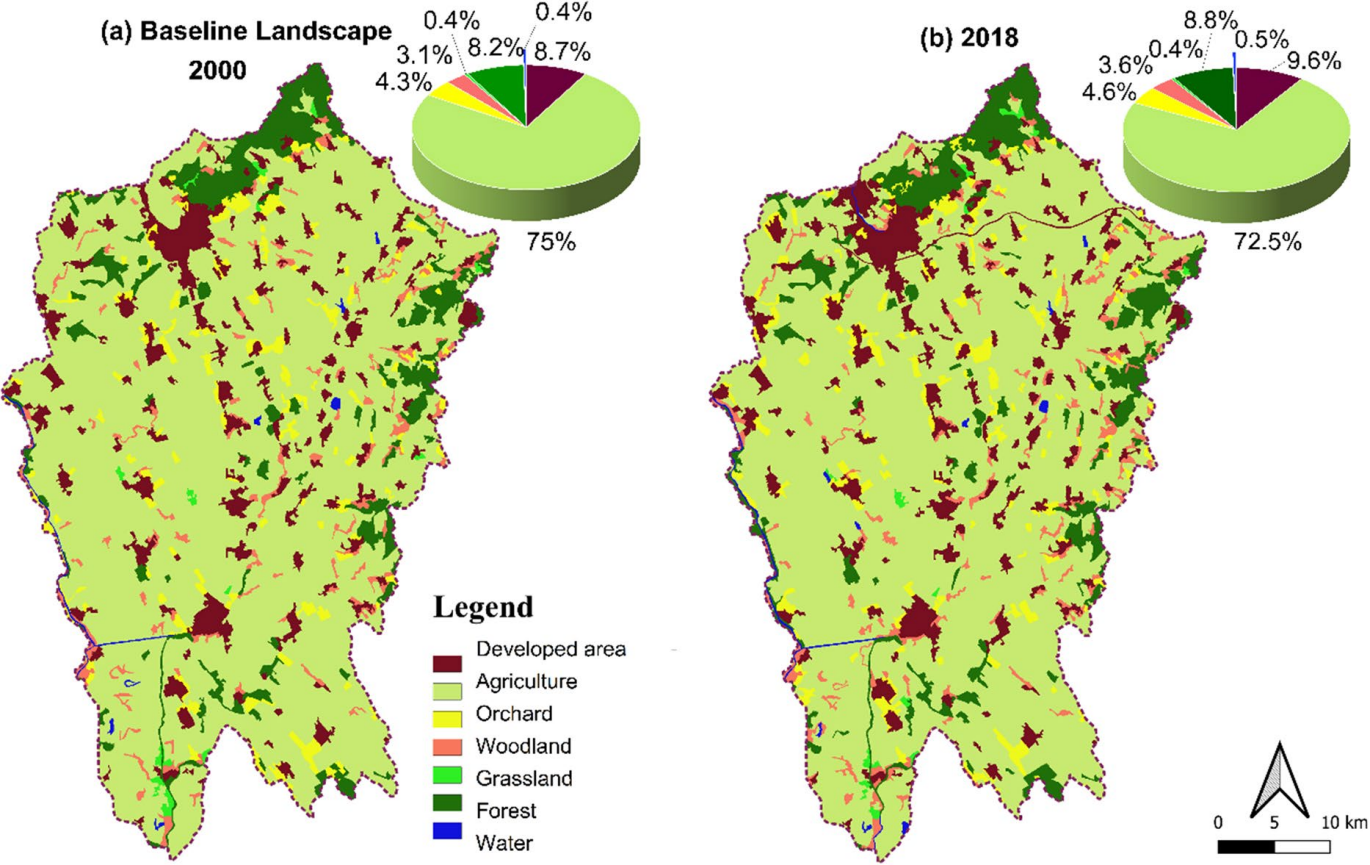
The spatial–temporal distribution of the LULC from 2000 to 2018 in the Nitra region, Slovakia

**Fig. 4:**
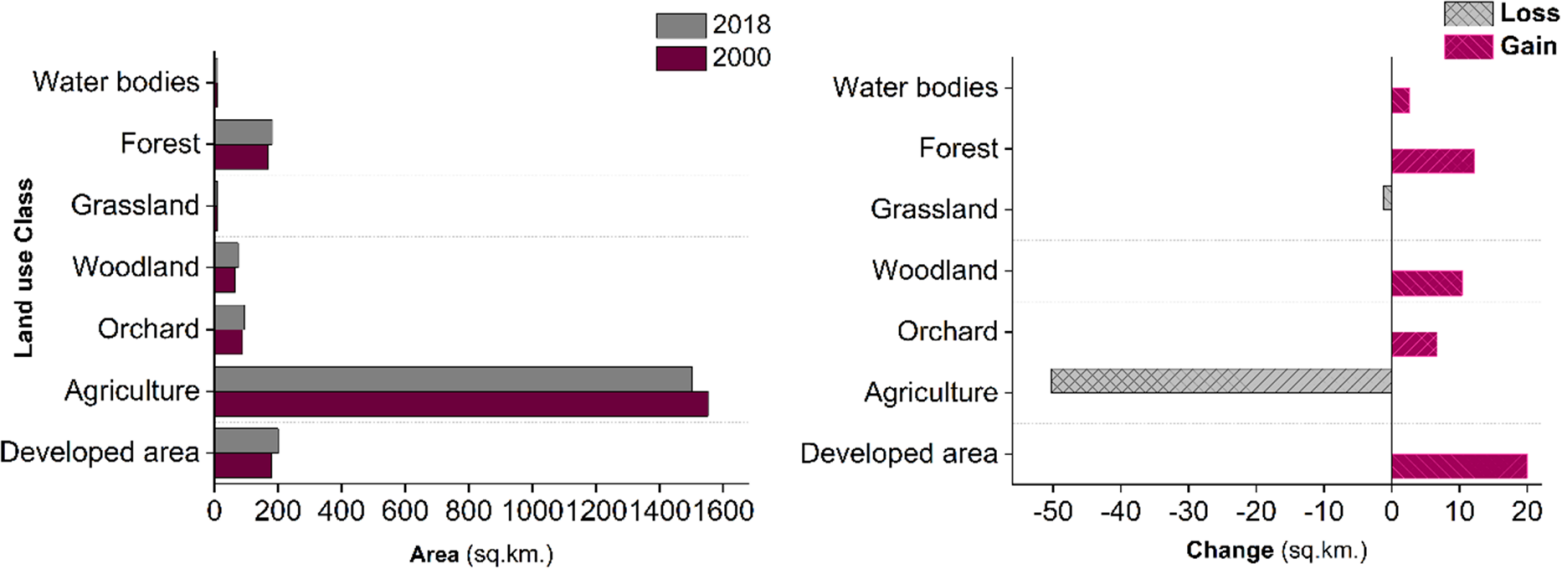
Land use change in the study area

From 2000 to 2018, the land use/land cover of Nitra region changed substantially in response to urban construction (Fig. 3). Forests, water bodies, woodlands, and orchards all increased, while cultivated land and grasslands decreased (Fig. 2). In 2000, 75% of Nitra's total area was cultivated land. By 2018, this had decreased to 72.5%, due to increased concentration around cities and their surroundings. During the same period, urbanized space increased from 8.7% to 9.6%. Forest, woodland, and orchards grew by 20 km2, 13 km2, and 11 km2, respectively. Cultivated land and grasslands decreased by 50 km2 and 1.5 km2, respectively (Fig. 4).

### Climate data: precipitation and temperature

**Fig. 5:**
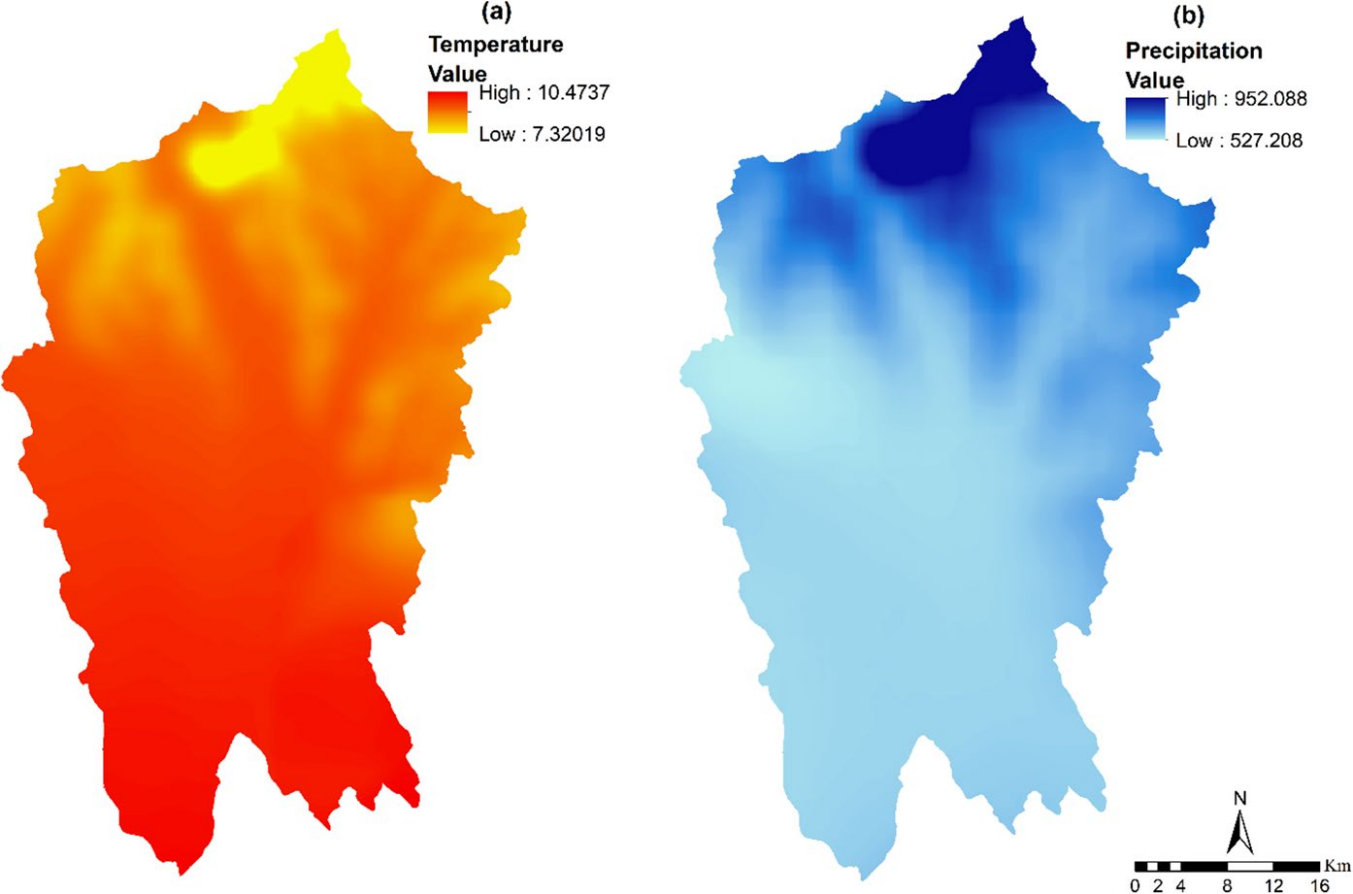
Temperature (**a**) and Precipitation (**b**) map for 2000

The Nitra region experienced a mean annual temperature of 7.32—10.47 °C and annual precipitation of 527.20—952.08 mm in 2000. The spatial pattern of both climate parameters was predominantly determined by elevation, with the southern and northern areas (Fig. 5a, b).

**Fig. 6:**
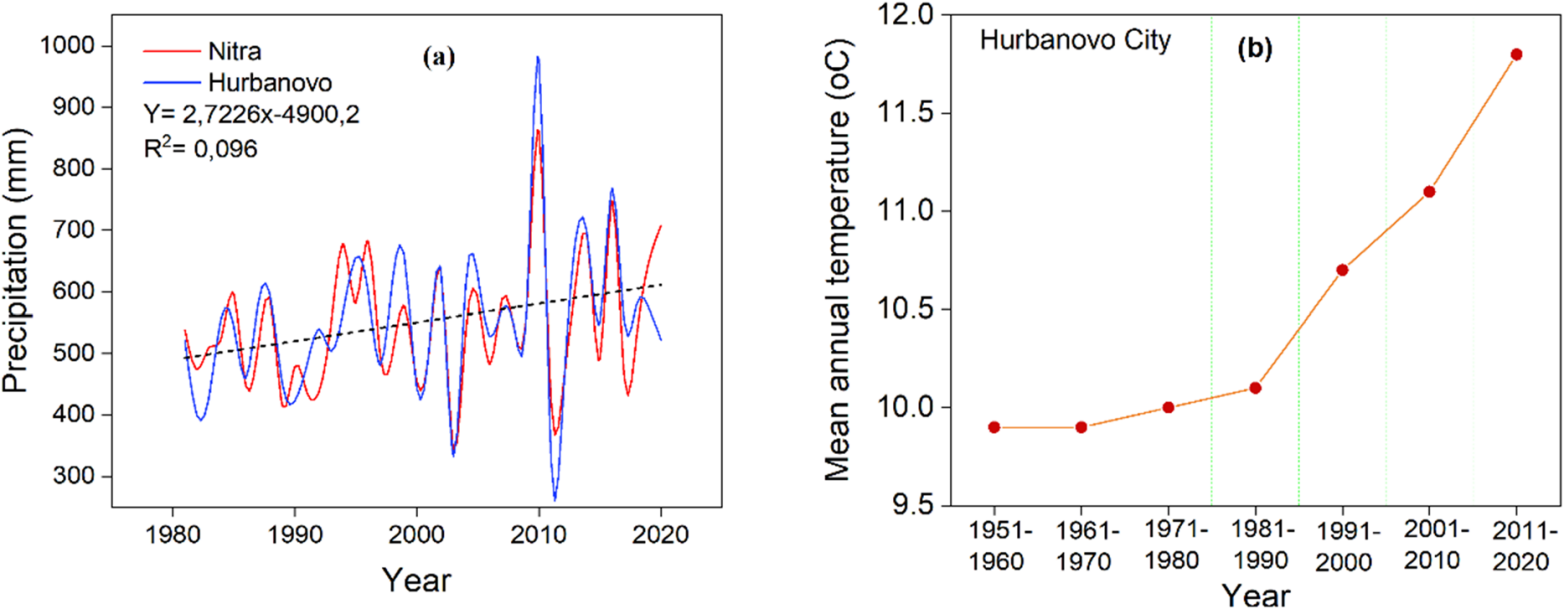
Long-term observed precipitation amount and mean temperature and their trends in the research area from 1981–2020 (*Source*: Slovak Hydrometeorological Institute data)

Figure 6a depicts the measured precipitation values in the Nitra region (Hurbanovo and Nitra climate station) over the 1981–2020 period. Analysis of the precipitation data for the period spanning 2000 to 2018 indicates a slight increase in the trend of precipitation. By plotting a trendline and calculating the overall increase in precipitation from 2000 to 2018, it is found that the total increment is approximately 25 mm.

### Ecosystem services change

#### Nutrient delivery ratio

**Fig. 7:**
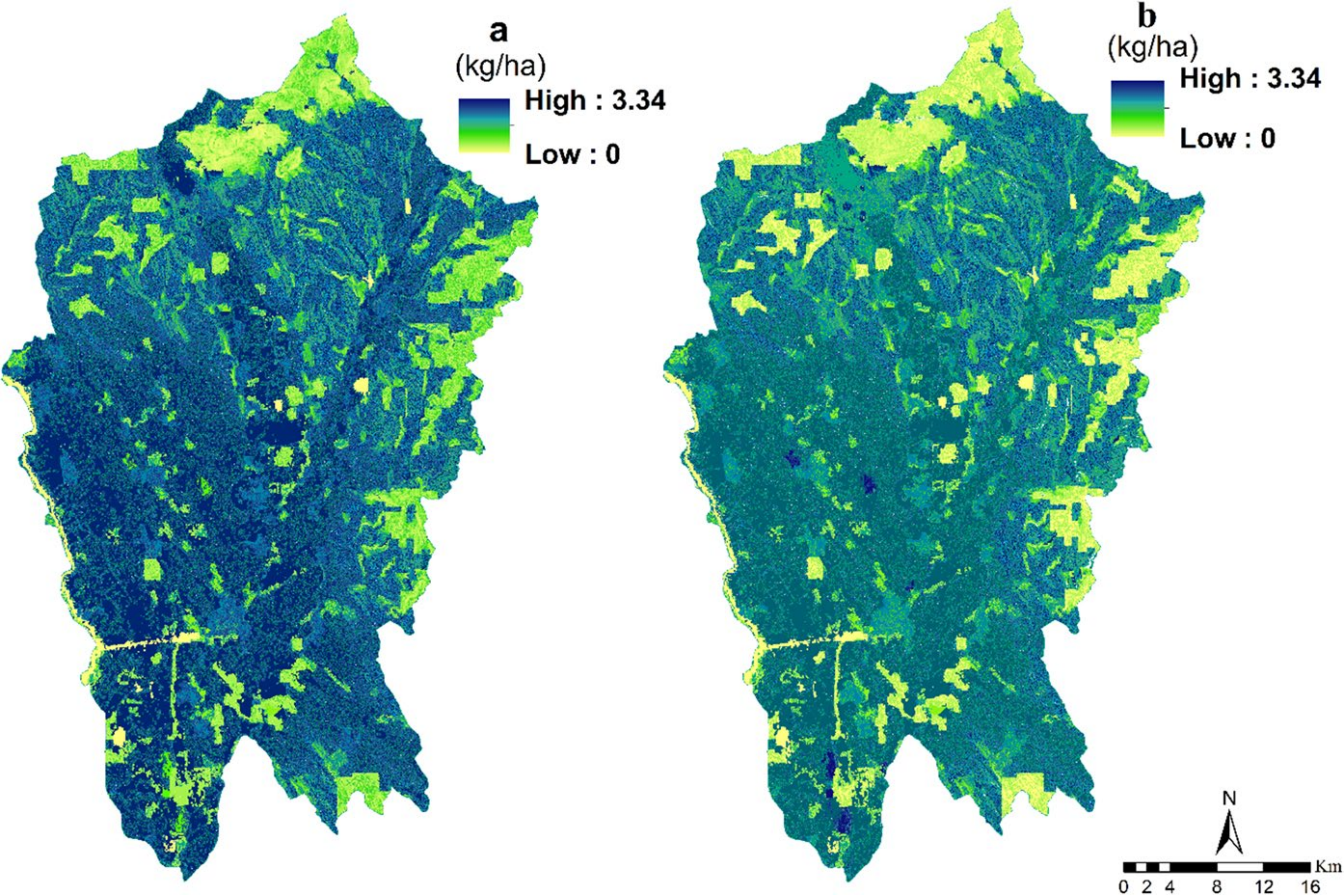
Spatial distribution and changes in nitrogen export (kg/ha) from 2000 to 2018 (**a**: nitrogen export in 2000; **b**: nitrogen export in 2018)

The Nutrient Delivery Ratio model was used to analyze the source and transport processes of nutrients in the Nitra region of Slovakia. Results (Fig. 7) showed a trend of decreasing nitrogen transport, from the mean value of 2197.04 kg/ha in 2000 to 1980.32 kg/ha in 2018. Agricultural land had the highest nutrient export capacity (mean values of 15.63 kg/ha in 2000 and 15.57 kg/ha in 2018), while forest land had the lowest nutrient export capacity (mean values of 2.36 kg/ha in 2000 and 2.33 kg/ha in 2018).

### Soil retention

**Fig. 8:**
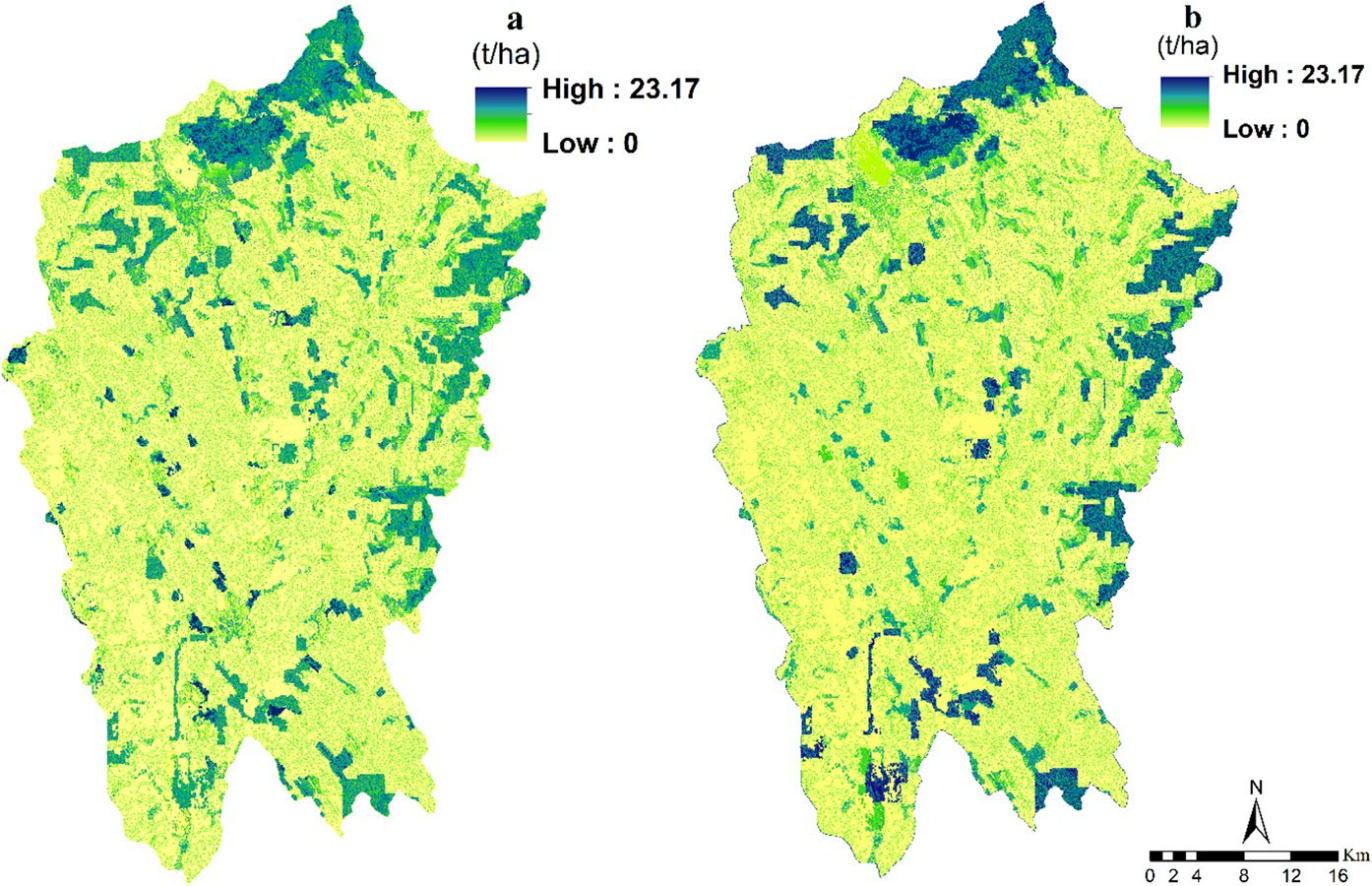
Spatial distribution and changes in soil retention (t/ha) from 2000 to 2018 (**a**: soil retention in 2000; **b**: soil retention in 2018)

The water-holding capacity of soils in the Nitra region has increased slightly, providing essential sustenance for life and supporting the functioning of ecosystems. The mean soil retention increased significantly from 0.48 million tons in 2000 to 0.76 million tons in 2018, which can be largely attributed to the expansion of forest area. The topography of a given area has a significant effect on soil retention capacity. In 2000, forest land had the highest soil retention capacity on average, at 4.80 t/ha. This value further increased to 5.54 t/ha in 2018. Mapping soil retention capacity revealed that forest areas were significantly associated with higher values of this ES than agricultural land. The mean value of soil retention capacity was 3.41 t/ha in 2000 and 3.02 t/ha in 2018 for agricultural land, whereas the mean value of soil retention capacity in forest land was higher. This study suggests that soil retention capacity was lower in agricultural lands compared to forest lands in the Nitra region, likely a consequence of intense agricultural activity. Though there was a general rise in soil retention amounts, certain parts of the Nitra region were subject to declines in soil retention owing to increased urban development (Fig. 8).

### Carbon storage

**Fig. 9:**
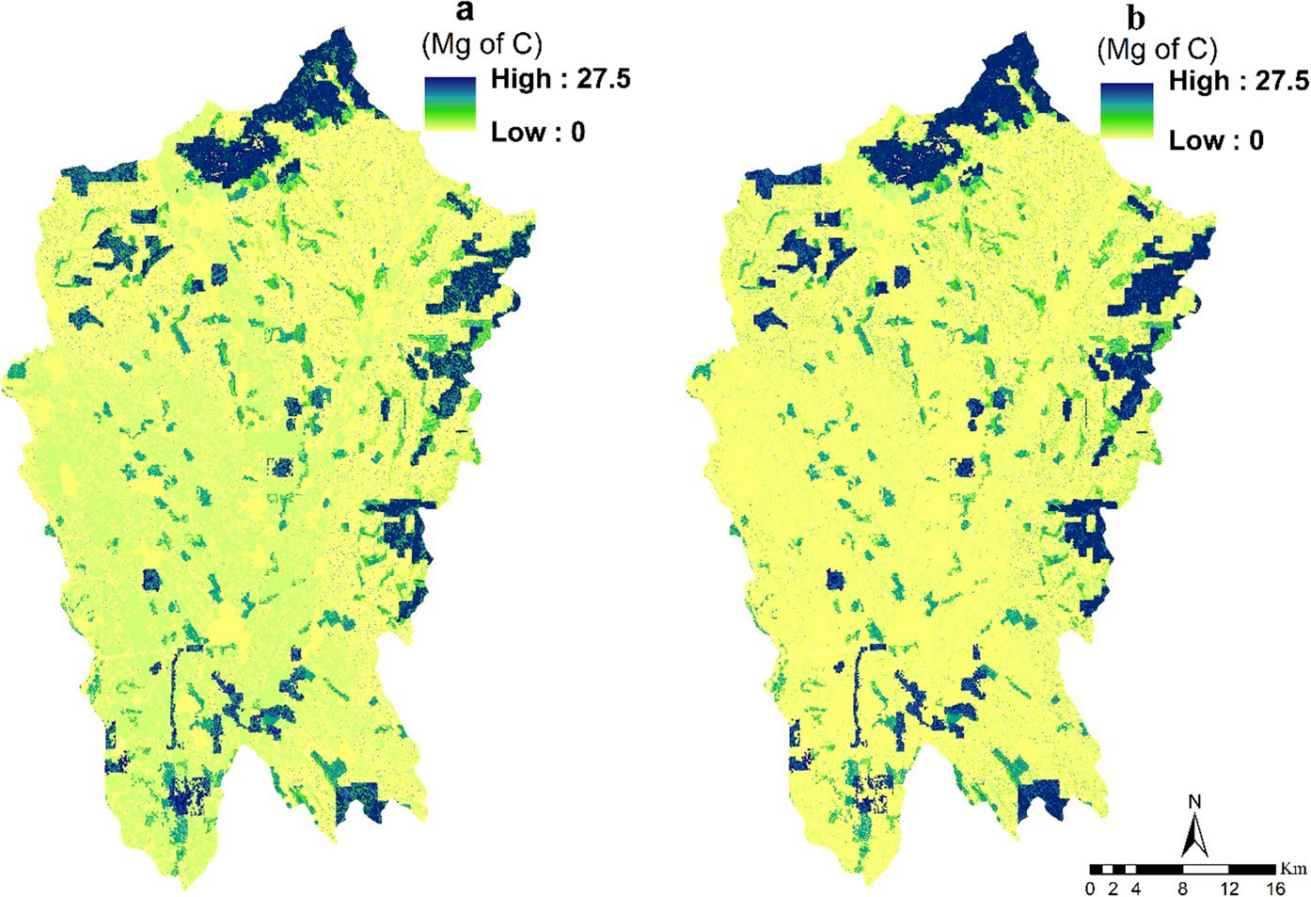
Spatial distribution and changes of carbon storage (Mg/ha) from 2000 to 2018 (**a**: carbon storage in 2000; **b**: carbon storage in 2018)

Carbon storage is one of the most vital ES that reduce atmospheric CO_2_ which accelerates climate change. The total amount of carbon storage in the research area was found to increase from 11,714,261 Mg C in 2000 to 12,397,747 Mg C in 2018 (at a rate of 5.8%). While the overall trend reflected an increase in carbon storage, some areas in the Nitra region showed a decrease in carbon storage (Fig. 9).

### Water yield

**Fig. 10:**
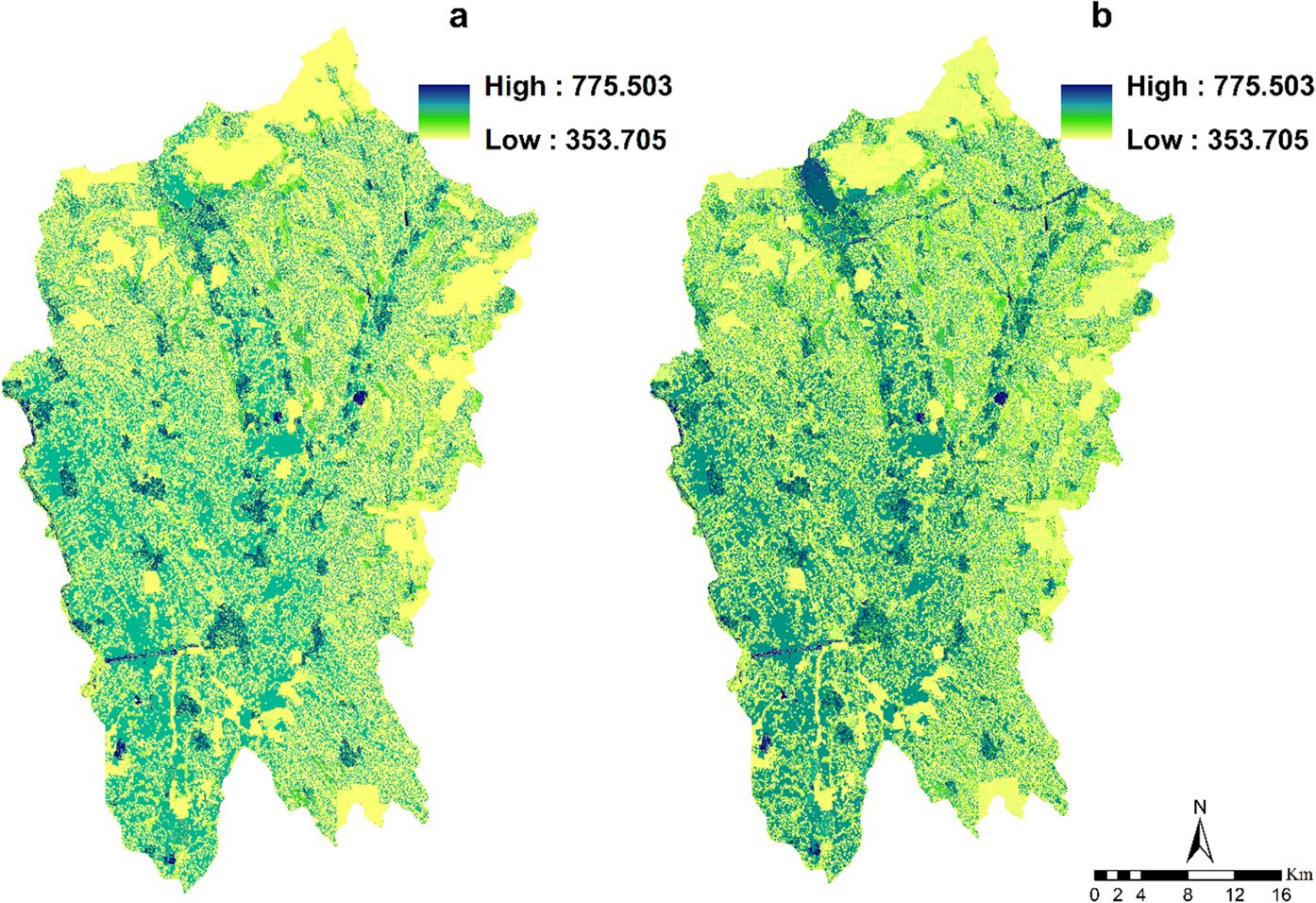
Spatial distribution and changes in water yield (m3/ha) from 2000 to 2018 (**a**: water yield in 2000; **b**: water yield in 2018)

Water yield depicts the potential of fresh water for hydropower generation, food production and drinking water. The mean amount of water yield for the region of Nitra was 1.38 billion m^3^ in 2000 reaching 2.05 billion m^3^ in 2018 (Fig. 10). Generally, water yield increased over time as a result of the expansion of developed areas and higher precipitation. In 2000 and 2018, forest land had the lowest water retention capacity, averaging 158 m3/ha and 285 m3/ha, respectively. This class thus served as a major hotspot for water retention services throughout the study period. Agricultural land had an elevated water yield capacity, with mean values of 253 m3/ha and 410 m3/ha in 2000 and 2018, respectively. The highest water yield capacity was found in urban areas, with 293 m3/ha and 473 m3/ha in 2000 and 2018, respectively.

### Analysis of the factor importance

**Fig. 11:**
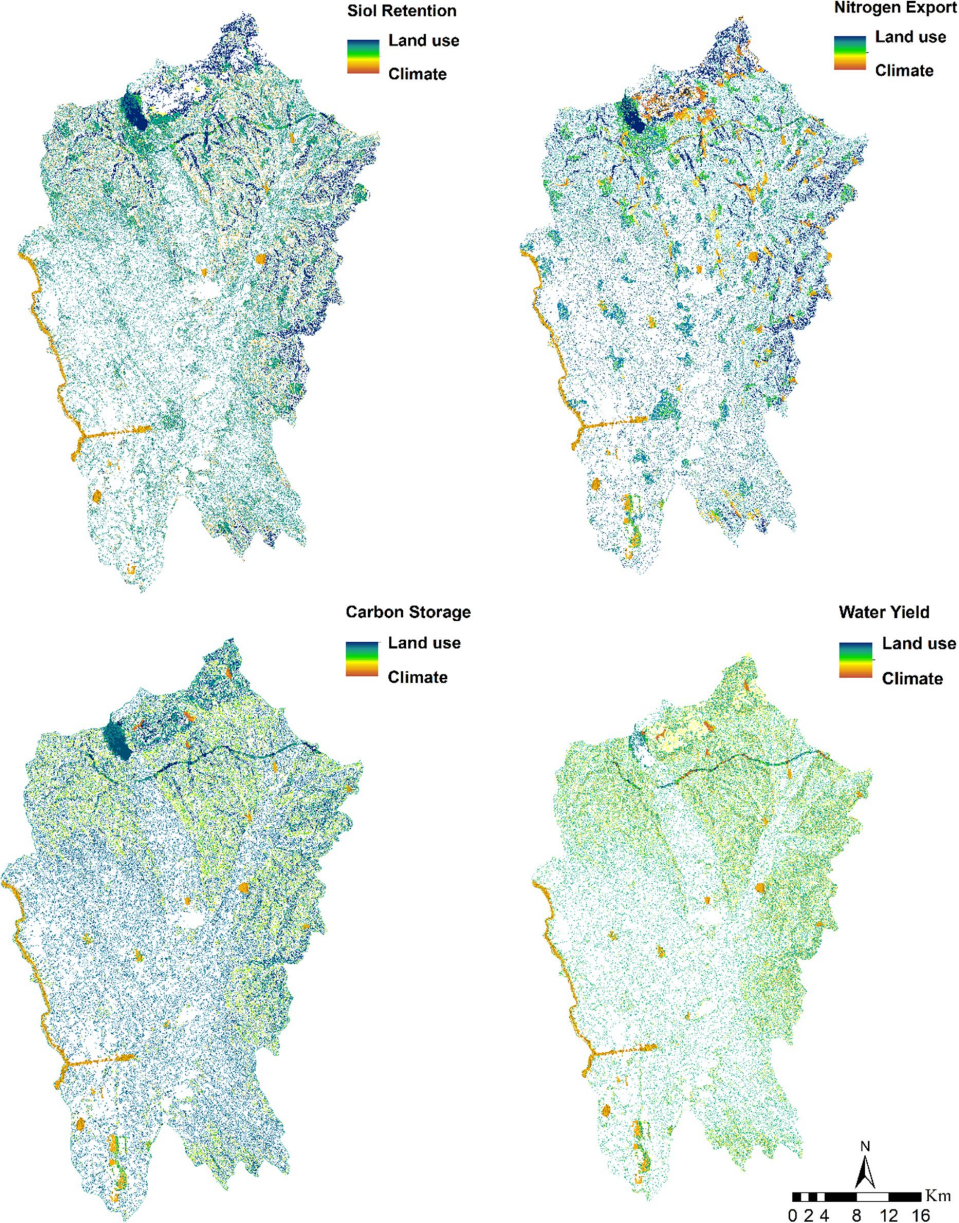
Land use importance—Climate importance on ecosystem services

A factor analysis of all pixels in the study area revealed that land use had a larger influence on nitrogen export relative to climate change, as attested by an RII of 60.03%. Moreover, soil retention and carbon storage, respectively, exhibited markedly distinct patterns, with land use being identified as a more influential factor than climate change in 59.11% and 83.01% of analyzed pixels (Fig. 11). The water yield model indicated that 58.21% of pixels experienced more significant effects from land use changes when compared to climatic factors. It is evident that land use change has a greater impact on ES than climate change; however, the latter should not be underestimated as it still has a noteworthy contribution. It is essential to factor in the impact of climate change when managing ES, as this phenomenon may induce both direct and indirect ecological alterations.

### Hotspot and coldspot analysis

**Fig. 12:**
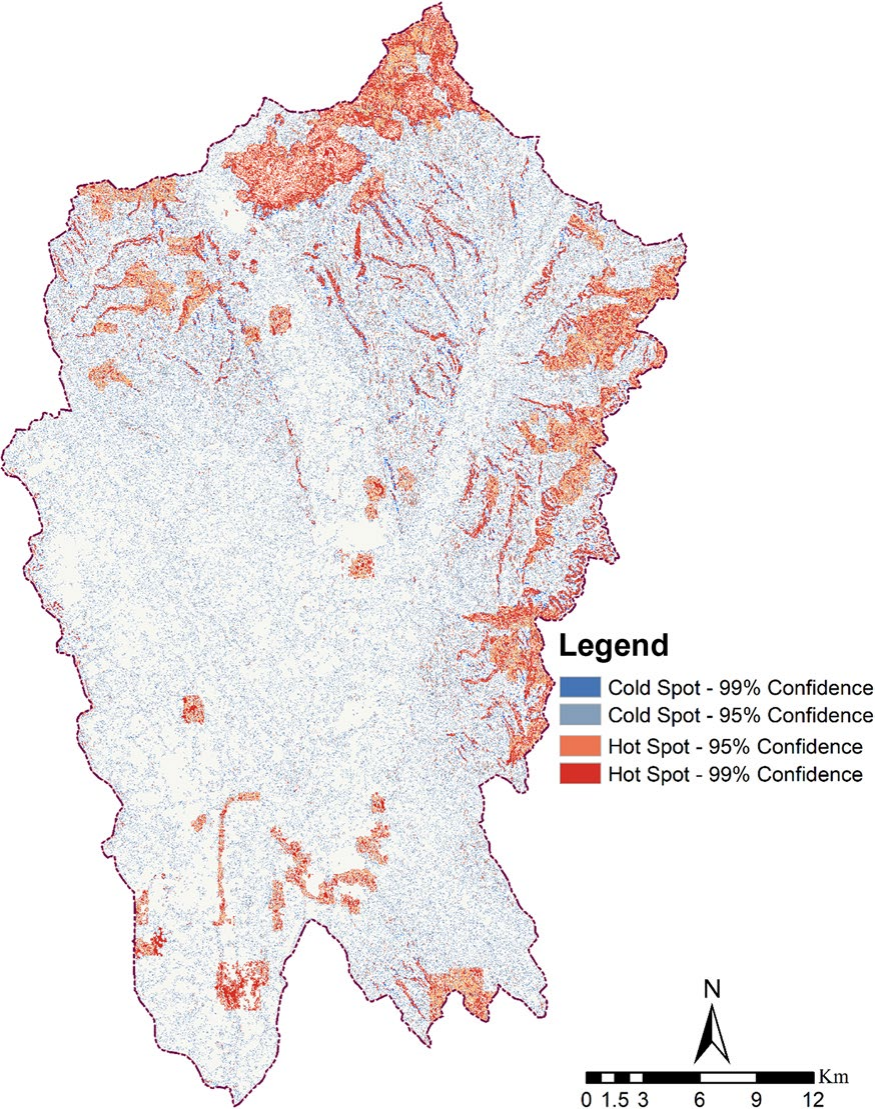
Spatial-explicitly mapping of the hotspots and coldspots

The key to ES assessment was to identify spatiotemporal dynamics and to identify areas of high and low ES provision (i.e., ecosystem services hotspots and coldspots). The statistically significant hotspots and coldspots with different confidence levels are shown in Fig. 12. Generally, P < 0.05 (i.e., 95% confidence level) is defined as statistically significant. During 2000–2018, the spatial–temporal changes and the area proportion of hotspots and coldspots were investigated. The forest areas upstream always show a concentrated distribution of hotspots of level 1. Most hotspot areas were located in the northern and eastern regions, whereas coldspots were observed in the central and southern regions across the study period. Approximately 14.62% of the studied ESs demonstrated statistically significant hotspot areas with the highest potential for providing services. In comparison, twice as much land is accounted for by notable coldspot areas (41.13%). Generally, coldspots offer one to two services of high significance and three to four services of low significance, while hotspots provide three to four services of high importance and two to three services of low importance, occupying 24% of the overall area.

## Discussion

### Land use and climate effect

The integration of ES evaluation and scenario analysis can yield reliable assessment of the relative importance of multiple drivers on ES. In this study, the RII was applied to distinguish which of the two drivers – climate and land use change – had more prominent effects on provision of selected ES. Results obtained indicated that climate and land use change, as well as their interactions, have significant influence on the ecosystem's capacity to provide ES.

Our results demonstrate that land use has a greater impact than climate change on soil retention and carbon storage at smaller spatial scales. This finding is supported by the previous studies (Gao & Wang, 2019; Scafidi & Gilfillan, 2019; Williams & Powers, 2019) which have also shown that land use change has a greater impact than climate change on these services. Additionally, land use change had a larger impact than climate change on soil export on a large scale (Adelisardou et al., 2021). It was discovered that land use had a greater impact on nutrient export than climate change. Surface and subsurface flow, as well as human-caused sources including industrial effluent, water treatment plant discharges, and fertilizer used in farming and residential areas, were identified as contributors of the nutrient movement (Sharp et al., 2016). Our results in terms of water yield service revealed a strong correlation between this service and land use change. Studies (Gao et al., 2017a, 2017b; Gao et al., 2017a, 2017b) demonstrated that the service of water yield was more strongly correlated to climate change than land use change. However, our results for the RII factor were not consistent with those of other studies. As mentioned in the section on data availability, our data for climate change in 2018 were based on a time-series of the precipitation and temperature change from operating climate stations (one proxy-value of change was set for the whole research area, the spatial aspect of climate parameters values remained the same as in 2000). Consequently, omitting updates on the spatial dimension of climatic data would affect the relative importance of ES drivers. Results from Central Nitra (cultivated landscape) and North Nitra (orchard landscape to forest landscape) revealed an increase in the effect of land use change on soil moisture retention and carbon sequestration and a decrease in the effect of land use change on nitrate export.

### Strategies and implications

The evaluation of climate and land use impacts on the delivery of ESs has identified areas of increased activity as well as those that are vulnerable to alterations. This information can be utilized to assist local administrators in the judicious deployment of available resources. Strategies such as wildlife conservation, ecological rehabilitation, and biodiversity safeguarding can be implemented to ensure the uninterrupted provision of ES. Consequently, through wise land utilization management, authorities can be confident when deciding how and where to prioritize investments to optimize the cost-effectiveness of enhancing or restoring ES in vulnerable sites (Peng et al., 2020; Lyu et al., 2018). Farmers and landholders in Nitra and other similar regions can be supported to reduce their use of synthetic fertilizers and shift toward more sustainable practices like crop rotation, which can help reduce soil degradation and nutrient runoff. Additionally, the introduction of modern agroforestry systems and the expansion of traditional farming techniques can result in a significant increase in carbon storage, biodiversity levels, and financial rewards. Moreover, enlarging resources in ES coldspots for the purpose of landscape utilization are essential. To improve cold spots, recreational activities ought to be facilitated through increased infrastructure and personnel, and services should be upgraded to make them more appealing to natives and visitors. Financial promotions like tax exemptions can be applied to allure business organizations to locate in cold spots.

Specifically, our ES distribution and change maps for the Nitra region indicate that greater results can be achieved concerning carbon sequestration and soil preservation by focusing on forest lands in the Northeastern Nitra regions. The nutrient export reduction can be facilitated by funneling measures into the Central and Southern Nitra areas.

These results bridge the science-policy-management gap with easy-to-use indicators for assessing land use and climate-based ES gains. Furthermore, our highly descriptive maps of soil and water-related ESs can provide insights for landscape administration and authoritative decisions (Figs. 11 and 12). These maps serve as a valid baseline for quantifying the changes in ESs resulting from land use and climate factors (Tolessa et al., 2017). According to our relative importance analysis, land use changes were significantly more prolific with regards to carbon retention rates (and slightly more effective for other examined ESs) than climate shifts, particularly in forestry-rich territories like in the North of Nitra region. From our study, we concluded that judicious land utilization can augment the potential of ecosystems to uphold soil and water-related ESs. Policy-makers ought to adopt and managers need to implement climate-adaptive measures cum management by recognizing areas of synergy for future conservation and those deemed for transformation.

## Conclusions

This study presents a comprehensive spatial analysis of soil- and water-related ES, exploring the historical and interactive impacts of land use and climate change in the Nitra region, Slovakia. The relative contributions of land use and climate change to shifts in the provision of ES were explored. Furthermore, areas of either diminished or enhanced ecological functionality were identified. Results indicated that land use modifications had a more pronounced effect than climate change on carbon storage, as well as other ES such as soil retention, water yield, and nutrient delivery ratio. The findings of this study have implications for theory, methods, and policy that suggest the use of ES information within landscape-level planning could improve the efficacy of ecosystem conservation strategies. Our study corroborates the hypothesis that climate-adaptive management can aid managers in designing plans that are spatially more comprehensive. The evaluation of the impacts of driving factors on ES can be enhanced through the use of a combination of scenario analysis, InVEST models, relative importance index, and hotspot-coldspots analysis.

These tools facilitate a better comprehension of the processes underlying changes in ES, leading to the formulation of effective strategies. This research can inform activities and policies that support sustainability in Nitra and other similar regions by elucidating where protections and investments should be made on the landscape scale.

## References

[CR1] Adelisardou F, Jafari HR, Malekmohammadi B, Minkina T, Zhao W, Karbassi A (2021). Impacts of land use and land cover change on the interactions among multiple soil-dependent ecosystem services (case study: Jiroft plain, Iran). Environmental Geochemistry and Health.

[CR2] Aronson, J., & van Andel, J. (2006). *Challenges for ecological theory* (pp. 223–233). Blackwell Publishing: Oxford, UK.

[CR3] Bao, Z., Shifaw, E., Deng, C., Sha, J., Li, X., Hanchiso, T., & Yang, W. (2022). Remote sensing-based assessment of ecosystem health by optimizing vigor-organization-resilience model: A case study in Fuzhou City, China. *Ecological Informatics*, *72*, 101889. 10.1016/j.ecoinf.2022.101889

[CR4] Chen, W., Chi, G., & Li, J. (2020). The spatial aspect of ecosystem services balance and its determinants. *Land Use Policy*, *90*, 104263. 10.1016/j.landusepol.2019.104263

[CR5] Chen, Y., Ma, Y., Pan, J., Zhang, S., Zhang, X., Wu, R., & Li, X. (2023). Integrating ecosystem health diagnosis into the construction of ecological security network—A case study in Qujing City, China. *Ecological Indicators*, *146*, 109780. 10.1016/j.ecolind.2022.109780

[CR6] Climate Atlas of Slovakia: https://climate-adapt.eea.europa.eu

[CR7] Cornell JD, Quintas-Soriano C, Running K, Castro AJ (2019). Examining concern about climate change and local environmental changes from an ecosystem service perspective in the Western U.S. Environmental Science and Policy.

[CR8] Costanza R, de Groot R, Braat L, Kubiszewski I, Fioramonti L, Sutton P, Farber S, Grasso M (2017). Twenty years of ecosystem services: How far have we come and how far do we still need to go?. Ecosystem Services.

[CR9] Gao J, Li F, Gao H, Zhou C, Zhang X (2017). The impact of land-use change on water-related ecosystem services: A study of the Guishui River Basin, Beijing, China. Journal of Cleaner Production.

[CR10] Gao, J., & Wang, L. (2019). Embedding spatiotemporal changes in carbon storage into urban agglomeration ecosystem management—A case study of the Yangtze River Delta, China. *Journal of Cleaner Production*, *237*, 117764. 10.1016/j.jclepro.2019.117764

[CR11] Gao J-M, Wu L, Chen Y-P, Zhou B, Guo J-S, Zhang K, Ouyang W-J (2017). Spatiotemporal distribution and risk assessment of organotins in the surface water of the Three Gorges Reservoir Region, China. Chemosphere.

[CR13] Hekmatfard M, Farahmand F, Ebrahimi I (2013). Effects of prosthetic mass distribution on the spatiotemporal characteristics and knee kinematics of transfemoral amputee locomotion. Gait & Posture.

[CR14] Howe C, Suich H, Vira B, Mace GM (2014). Creating win-wins from trade-offs? Ecosystem services for human well-being: A meta-analysis of ecosystem service trade-offs and synergies in the real world. Global Environmental Change.

[CR15] Lang Y, Song W (2019). Quantifying and mapping the responses of selected ecosystem services to projected land use changes. Ecological Indicators.

[CR16] Leal Filho, W., Azeiteiro, U. M., Balogun, A.-L., Setti, A. F. F., Mucova, S. A. R., Ayal, D., Totin, E., Lydia, A. M., Kalaba, F. K., & Oguge, N. O. (2021). The influence of ecosystems services depletion to climate change adaptation efforts in Africa. *Science of The Total Environment*, *779*, 146414. 10.1016/j.scitotenv.2021.146414 10.1016/j.scitotenv.2021.146414 33735656

[CR17] Lheureux, A., David, V., del Amo, Y., Soudant, D., Auby, I., Bozec, Y., Conan, P., Ganthy, F., Grégori, G., Lefebvre, A., Leynart, A., Rimmelin-Maury, P., Souchu, P., Vantrepote, V., Blondel, C., Cariou, T., Crispi, O., Cordier, M.-A., Crouvoisier, M., … Savoye, N. (2023). Trajectories of nutrients concentrations and ratios in the French coastal ecosystems: 20 years of changes in relation with large-scale and local drivers. *Science of The Total Environment*, *857*, 159619. 10.1016/j.scitotenv.2022.159619 10.1016/j.scitotenv.2022.159619 36280086

[CR18] Li F, Liu X, Zhang X, Zhao D, Liu H, Zhou C, Wang R (2017). Urban ecological infrastructure: An integrated network for ecosystem services and sustainable urban systems. Journal of Cleaner Production.

[CR19] Li, J., Zhang, C., & Zhu, S. (2021). Relative contributions of climate and land-use change to ecosystem services in arid inland basins. *Journal of Cleaner Production*, *298*, 126844. 10.1016/j.jclepro.2021.126844

[CR20] Li Y, Zhang L, Yan J, Wang P, Hu N, Cheng W, Fu B (2017). Mapping the hotspots and coldspots of ecosystem services in conservation priority setting. Journal of Geographical Sciences.

[CR21] Luiza Petroni, M., Siqueira-Gay, J., & Lucia Casteli Figueiredo Gallardo, A. (2022). Understanding land use change impacts on ecosystem services within urban protected areas. *Landscape and Urban Planning*, *223*, 104404. 10.1016/j.landurbplan.2022.104404

[CR22] Lyu R, Zhang J, Xu M, Li J (2018). Impacts of urbanization on ecosystem services and their temporal relations: A case study in Northern Ningxia, China. Land Use Policy.

[CR23] Ma, J., Ding, X., Shu, Y., & Abbas, Z. (2022a). Spatio-temporal variations of ecosystem health in the Liuxi River Basin, Guangzhou, China. *Ecological Informatics*, *72*, 101842. 10.1016/j.ecoinf.2022.101842

[CR24] Ma, S., Li, Y., Zhang, Y., Wang, L.-J., Jiang, J., & Zhang, J. (2022b). Distinguishing the relative contributions of climate and land use/cover changes to ecosystem services from a geospatial perspective. *Ecological Indicators*, *136*, 108645. 10.1016/j.ecolind.2022.108645

[CR25] Manes, S., Vale, M. M., Malecha, A., & Pires, A. P. F. (2022). Nature-based solutions promote climate change adaptation safeguarding ecosystem services. *Ecosystem Services*, *55*, 101439. 10.1016/j.ecoser.2022.101439

[CR26] Nijhum, F., Westbrook, C., Noble, B., Belcher, K., & Lloyd-Smith, P. (2021). Evaluation of alternative land-use scenarios using an ecosystem services-based strategic environmental assessment approach. *Land Use Policy*, *108*, 105540. 10.1016/j.landusepol.2021.105540

[CR27] Oh, S., & Yoon, Y. (2022). Data-driven risk analysis of unmanned aircraft system operations considering spatiotemporal characteristics of population distribution. *Transportation Research Interdisciplinary Perspectives*, *16*, 100732. 10.1016/j.trip.2022.100732

[CR28] Peng, J., Tian, L., Zhang, Z., Zhao, Y., Green, S. M., Quine, T. A., Liu, H., & Meersmans, J. (2020). Distinguishing the impacts of land use and climate change on ecosystem services in a karst landscape in China. *Ecosystem Services*, *46*, 101199. 10.1016/j.ecoser.2020.101199

[CR29] Postigo JC (2021). Navigating capitalist expansion and climate change in pastoral social-ecological systems: Impacts, vulnerability and decision-making. Current Opinion in Environmental Sustainability.

[CR30] Qiao, W., & Huang, X. (2022). The impact of land urbanization on ecosystem health in the Yangtze River Delta urban agglomerations, China. *Cities*, *130*, 103981. 10.1016/j.cities.2022.103981

[CR31] Qiu, J., Yu, D., & Huang, T. (2022). Influential paths of ecosystem services on human well-being in the context of the sustainable development goals. Science of The Total Environment, 852, 158443. 10.1016/j.scitotenv.2022.158443 10.1016/j.scitotenv.2022.158443 36055481

[CR32] QGIS Development Team (YEAR). QGIS Geographic Information System. Open-Source Geospatial Foundation Project. http://qgis.osgeo.org

[CR33] Reed MS, Stringer LC, Dougill AJ, Perkins JS, Atlhopheng JR, Mulale K, Favretto N (2015). Reorienting land degradation towards sustainable land management: Linking sustainable livelihoods with ecosystem services in rangeland systems. Journal of Environmental Management.

[CR34] Saeed, U., Arshad, M., Hayat, S., Morelli, T. L., & Ali Nawaz, M. (2022). Analysis of provisioning ecosystem services and perceptions of climate change for indigenous communities in the Western Himalayan Gurez Valley, Pakistan. *Ecosystem Services*, *56*, 101453. 10.1016/j.ecoser.2022.101453

[CR35] Scafidi, J., & Gilfillan, S. M. V. (2019). Offsetting Carbon Capture and Storage costs with methane and geothermal energy production through reuse of a depleted hydrocarbon field coupled with a saline aquifer. *International Journal of Greenhouse Gas Control*, *90*, 102788. 10.1016/j.ijggc.2019.102788

[CR36] Sharp, R., Tallis, H. T., Ricketts, T., Guerry, A. D., Wood, S. A., Chaplin-Kramer, R., Nelson, E., Ennaanay, D., Wolny, S., Olwero, N., Vigerstol, K., Pennington, D., Mendoza, G., Aukema, J., Foster, J., Forrest, J., Cameron, D., Arkema, K., Lonsdorf, E., Kennedy, C., Verutes, G., Kim, C. K., Guannel, G., Papenfus, M., Toft, J., Marsik, M., Bernhardt, J., Griffin, R., Glowinski, K., Chaumont, N., Perelman, A., Lacayo, M., Mandle, L., Hamel, P., Vogl, A. L., Rogers, L., Bierbower. W. (2016). In EST 3.3.0 User's Guide. The Natural Capital Project, Stanford University, University of Minnesota, The Nature Conservancy and World Wildlife Fund, Stanford.

[CR37] Song B, Yi S, Jia H, Nahm W-H, Kim J-C, Lim J, Lee J-Y, Sha L, Mao L, Yang Z, Nakanishi T, Hong W, Li Z (2018). Pollen record of the mid- to late-Holocene centennial climate change on the East coast of South Korea and its influential factors. Journal of Asian Earth Sciences.

[CR38] Su, G., Zhang, S., Hu, M., Yao, W., Li, Z., & Xi, Y. (2022). The modified layer-by-layer weakening solar radiation models based on relative humidity and air quality index. *Energy*, *239*, 122488. 10.1016/j.energy.2021.122488

[CR39] Tolessa T, Senbeta F, Kidane M (2017). The impact of land use/land cover change on ecosystem services in the central highlands of Ethiopia. Ecosystem Services.

[CR40] Wang X, Dong X, Liu H, Wei H, Fan W, Lu N, Xu Z, Ren J, Xing K (2017). Linking land use change, ecosystem services and human well-being: A case study of the Manas River Basin of Xinjiang, China. Ecosystem Services.

[CR41] Ward M, Possingham H, Rhodes JR, Mumby P (2018). Food, money and lobsters: Valuing ecosystem services to align environmental management with Sustainable Development Goals. Ecosystem Services.

[CR42] Weerahewa, J., Timsina, J., Wickramasinghe, C., Mimasha, S., Dayananda, D., & Puspakumara, G. (2023). Ancient irrigation systems in Asia and Africa: Typologies, degradation and ecosystem services. *Agricultural Systems*, *205*, 103580. 10.1016/j.agsy.2022.103580

[CR43] Weiskopf, S. R., Rubenstein, M. A., Crozier, L. G., Gaichas, S., Griffis, R., Halofsky, J. E., Hyde, K. J. W., Morelli, T. L., Morisette, J. T., Muñoz, R. C., Pershing, A. J., Peterson, D. L., Poudel, R., Staudinger, M. D., Sutton-Grier, A. E., Thompson, L., Vose, J., Weltzin, J. F., & Whyte, K. P. (2020). Climate change effects on biodiversity, ecosystems, ecosystem services, and natural resource management in the United States. *Science of The Total Environment*, *733*, 137782. 10.1016/j.scitotenv.2020.137782 10.1016/j.scitotenv.2020.137782 32209235

[CR44] Williams NG, Powers MD (2019). Carbon storage implications of active management in mature Pseudotsuga menziesii forests of western Oregon. Forest Ecology and Management.

[CR45] Zhang, X., Yang, H., Zhang, W., Fenicia, F., Peng, H., & Xu, G. (2022a). Hydrologic impacts of cascading reservoirs in the middle and lower Hanjiang River basin under climate variability and land use change. *Journal of Hydrology: Regional Studies*, *44*, 101253. 10.1016/j.ejrh.2022.101253

[CR46] Zhang, Y., Wu, T., Song, C., Hein, L., Shi, F., Han, M., & Ouyang, Z. (2022b). Influences of climate change and land use change on the interactions of ecosystem services in China’s Xijiang River Basin. *Ecosystem Services*, *58*, 101489. 10.1016/j.ecoser.2022.101489

[CR47] Zope PE, Eldho TI, Jothiprakash V (2016). Impacts of land use–land cover change and urbanization on flooding: A case study of Oshiwara River Basin in Mumbai, India. CATENA.

[CR48] Zou, S., Qian, J., Xu, B., Tu, Z., Zhang, W., Ma, X., & Liang, Y. (2022). Spatiotemporal changes of ecosystem health and their driving mechanisms in alpine regions on the northeastern Tibetan Plateau. *Ecological Indicators*, *143*, 109396. 10.1016/j.ecolind.2022.109396

